# A conceptual framework to facilitate the mental health of student nurses working with persons with intellectual disabilities

**DOI:** 10.4102/curationis.v38i1.1481

**Published:** 2015-11-25

**Authors:** Elsie S. Janse van Rensburg, Marie Poggenpoel, Chris Myburgh

**Affiliations:** 1 Department of Nursing Science, University of Pretoria, South Africa; 2 Department of Nursing Science, University of Johannesburg, South Africa; 3 Department of Educational Psychology, University of Johannesburg, South Africa

## Abstract

**Background:**

Student nurses (SNs) experience emotional discomfort during placement in the clinical psychiatric learning environment. This may negatively influence their mental health. Limited support is available to assist both SNs working with persons with intellectual disabilities and nurse educators during clinical accompaniment.

**Objectives:**

This article aims to discuss the generation of this framework to enhance student support.

**Method:**

A theory-generative, qualitative, exploratory, descriptive, contextual design was utilised to develop the framework by applying four steps. In step 1 concept analysis identified the central concept through field work. Data were collected from 13 SNs purposively selected from a specific higher educational institution in Gauteng through two focus group interviews, reflective journals, a reflective letter, naïve sketches, drawings and field notes and analysed with thematic coding. The central concept was identified from the results, supported by a literature review and defined by essential attributes. The central concept was classified through a survey list and demonstrated in a model case. In step 2 the central concepts were placed into relationships with each other. The conceptual framework was described and evaluated in step 3 and guidelines for implementation were described in step 4. The focus of this article will be on generating the conceptual framework.

**Results:**

The central concept was ‘the facilitation of engagement on a deeper emotional level of SNs’. The conceptual framework was described and evaluated.

**Conclusion:**

The conceptual framework can enhance the educational practices of nurse educators and can SN’s practices of care for persons with intellectual disabilities.

## Introduction

In this article the preferred term for individuals with mental retardation is ‘persons with intellectual disabilities’. According to Geiger (2012:1) data on the prevalence of children with severe intellectual disabilities in South Africa are very limited.

The context of this study involved persons with intellectual disabilities residing at a specific rehabilitation facility for persons with intellectual disabilities in Gauteng, South Africa. This facility had 120 residents at the time of data collection, many of whom were mentally and physically profoundly challenged children and adults in need of special or total care. Student nurses (SNs) from a specific higher educational institution were placed at this rehabilitation facility for 90 hours as a clinical psychiatric learning environment (Janse van Rensburg, Poggenpoel & Myburgh 2012:762). Limited structured support from nurse educators was available to SNs working in this clinical learning environment, which may increase their stress levels, limiting their ability to manage challenging behaviours resulting in less effective therapeutic interactions with persons with intellectual disabilities (Mutkins, Brown & Thorsteinsson 2011:508). SNs identified a need for support whilst working with persons with intellectual disabilities (Janse van Rensburg *et al.* 2012:768). A conceptual framework was developed to enhance support during the clinical learning experience by providing accompaniment for SNs working with persons with intellectual disabilities.

### Problem statement

Working with persons with intellectual disabilities involves a variety of challenges and can be an emotionally draining experience leading to feelings of anger and/or depression. Caregivers providing care to persons with intellectual disabilities may experience a decrease in self-esteem and feel overwhelmed, frustrated, ashamed, anxious, angry, isolated, exhausted and depressed whilst providing care to persons with intellectual disabilities (Chadwick *et al.* 2013:123–124; Wei *et al* 2012:1673). Ketola and Stein (2013:24) confirm that SNs experience emotional discomfort during their initial interactions with mental health care users (MHCUs), especially when there is limited support. Persons with intellectual disabilities often have challenges in communicating, depending on their disability. Their inability to communicate may thus increase the emotional discomfort experienced by SNs. Emotional discomfort can also result from challenges demanding involvement from these SNs and may include demands on a physical (providing total care) and emotional (demonstrating patience and commitment) level from SNs. As their emotional discomfort and feelings of hopelessness increase, their stress levels escalate.

Karimollahi (2012:744) confirms that SNs may experience stress associated with feeling unprepared for the clinical psychiatric learning environment. Luo and Wang (2009:7) link stress to mental illness, which can be viewed as someone’s inability to adjust to and cope with the demands in his or her environment; and the absence of physical, mental and social well-being (World Health Organization [WHO] 2013). Nurses working with persons with intellectual disabilities also raised the need for emotional support in this demanding working context (Galvin & Timmins 2010:733).

The nurse educator plays a vital role in preparing SNs for placement in the clinical psychiatric learning environment. This role includes being a competent and supportive role model who aims at creating a positive learning experience for students. Clinical accompaniment assists students with forming a nursing identity in the clinical environment (Walker *et al.* 2014:103–112). Therefore, the nurse educator involved in clinical accompaniment plays an important role in the facilitation of the mental health of SNs in terms of guidance in adjusting and coping in this environment.

## Aim of this study

The aim of this study was to develop and describe a conceptual framework for nurse educators to provide support and guidance to SNs to adjust and cope whilst working with persons with intellectual disabilities.

## Background

SNs are placed in the clinical learning environment to integrate theory and practice. The clinical learning environment consists of acute and chronic settings. During clinical accompaniment the nurse educator plays a role in demonstrating knowledge and skills and providing guidance and support to SNs. This article will focus on the aspect of mental retardation within the chronic setting of psychiatric nursing.

## Trends

Although there are programmes and models for clinical supervision to develop professional identity (Severinsson & Sand 2010:669–677), a gap exists for a conceptual framework that supports SNs working with persons with intellectual disabilities to enhance their professional and personal growth.

## Research objectives

To generate a conceptual framework to facilitate the mental health of SNs taking care of persons with intellectual disabilities.

### Definitions of key concepts

#### Intellectual disability

The terms ‘intellectual disability’ and ‘mental retardation’ as used in the literature refer to children and adults with an intelligence quotient (IQ) of below 70 and impairment in their functioning at a physical, social or interpersonal level, restricting scholarly functioning before the onset of 18 years (American Association on Intellectual and Developmental Disabilities 2013). ‘Intellectually disabled’ refers to patients diagnosed with mental retardation residing in a rehabilitation facility in Gauteng.

#### Student nurses

This term refers to a student ‘following a programme of study in a nursing education and training institution’ (DoH *Nursing Act* 33 of 2005:30). A ‘student nurse’ will refer to a student registered for a degree in nursing at a higher education institution in an urban area placed at a mental health care facility for persons with intellectual disabilities.

#### Nurse educators

This term refers to a registered nurse with a master’s degree in nursing science involved in the education of nurses at university level, registered under section 31(1) of the *Nursing Act* 33 of 2005 to practice nursing or midwifery (*Mosby Medical Dictionary* 2009; *Nursing Act* 33 of 2005).

In this article, a ‘nurse educator’ will refer to an educator involved in teaching the theory and practical aspects of psychiatric nursing to SNs at a higher education institution.

## Contribution to field

The conceptual framework can enhance the clinical accompaniment practices of nurse educators in the clinical learning environment of Psychiatric Nursing Science. Improved clinical accompaniment will enhance support for SNs whilst enriching their learning experience and in turn will improve their practices of care towards persons with intellectual disabilities.

### Literature review

A limited number of studies were conducted on the support/guidance needed by the SNs during clinical accompaniment. (Lernihan & Sweeney 2010:27–28). There is a high level of stress, depression and burnout among support staff of persons with intellectual disabilities (Mutkins *et al.* 2011:500–502). A gap in literature was identified on the support for SNs working with persons with intellectual disabilities to enhance their professional identity and growth and improve their practices of care. Sandhu *et al.* (2012:315) support this by indicating that clinical supervision should include more than the content and theory; it should also focus on psychological and emotional support. The conceptual framework was generated to enhance psychological support during clinical accompaniment by nurse educators resulting in the facilitation of the mental health of SNs working with persons with intellectual disabilities.

## Research method and design

A qualitative, exploratory, descriptive, contextual and theory-generative design was used. The conceptual framework was developed utilising the steps of theory generation as proposed by Dickoff, James and Wiedenbach (1968:431–435) and Chinn and Kramer (2014:loc 596). The steps include (1) concept analysis, (2) the placing of the concepts in context, (3) a description of the conceptual framework, and (4) guidelines to operationalise the conceptual framework.

### Step 1: Concept analysis

Concept analysis first involved the identification of the central concept by means of fieldwork. Concept analysis will reflect the population and setting, data collection, data analysis, ethical considerations and trustworthiness.

## Population and sampling

The target population included 31 SNs in their final year of the four-year Baccalaureus Curationis Nursing Science degree at a higher educational institution in Gauteng. Purposive sampling was used to select participants who met the selection criteria of a 90-hour placement at one specific rehabilitation facility, and who were either English or Afrikaans-speaking. All the fourth-year B Cur Nursing Science degree students (31 students in total) were invited to participate, as they had previously been placed at the rehabilitation facility for persons with intellectual disabilities. A total of 13 participants (12 females and 1 male) chose to participate in the study.

## Data collection

Data were collected through focus group interviews, naïve sketches, drawings, reflective journals, a reflective letter, and field notes for data triangulation, ensuring that in-depth data were collected. The researcher informed participants about the aim and objectives of the study and informed consent was obtained before data collection was done.

The naïve sketches, drawings and field notes were collected on the same day. The naïve sketches and drawings were compiled by the SNs before the start of the focus group interview. The researcher asked the SNs to draw and write about their experiences of working with persons with intellectual disabilities. The reflective journals were kept by SNs whilst they were working with the persons with intellectual disabilities as part of the practical component of the module. The reflective letter was written by the students to the family of a mentally challenged individual whilst they were working at the facility.

Two focus groups’ interviews (one with Afrikaans and another with English-speaking participants) were facilitated by the researcher and a research assistant to explore the experience of these SNs. The focus group interviews were compiled according to the preferred languages to provide the participants with the opportunity to express themselves clearly in the language they felt comfortable with. The Afrikaans-speaking group interview consisted of seven members, whilst the English-speaking group consisted of six members. Focus groups’ interviews were conducted on separate dates in a venue at the higher educational institution where SNs studied to enhance accessibility to the venue. Participants were interviewed by the researcher and the question was: ‘Tell me about your experience working with persons with intellectual disabilities.’ The focus groups were audio-taped and verbatim transcriptions of the interviews were made with the written permission of participants. Data were collected from the SNs during Phase 1 (Janse van Rensburg *et al.* 2012:762–763) and used to generate a conceptual framework to facilitate the mental health of SNs working with persons with intellectual disabilities during Phase 2 of the study.

## Data treatment of step one

Focus groups’ interviews were transcribed verbatim. Data analysis for the focus groups’ interviews, naïve sketches, reflective journals and the reflective letter was done according to Tesch’s method of thematic coding (in Creswell 2009:186). Themes were categorised into major themes and sub-themes that supported a central storyline. An independent co-coder was included in the data analysis process to enhance trustworthiness and create a paper trail (Yin 2009:98). A consensus discussion was reached between the researcher and the independent coder on the findings of the research (Janse van Rensburg *et al.* 2012:764).

The central concept was identified from the results of the fieldwork based on the experiences of the SNs working with these individuals, and a literature review. This central concept is: SNs described working with mentally challenged individuals as a process of engagement on a deeper emotional level with these individuals. Concept analysis of these concepts was performed by using both dictionary and subject definitions to clarify conceptual meanings for the main concepts of this study. Various dictionaries, subject textbooks, internet sites (Medline, EBSCOhost), journals and peer-reviewed journals were explored to generate an understanding of the main concepts identified.

A list of essential and related attributes for the concepts in the central concept was compiled to identify, analyse and synthesise the attributes for the definition of the central concept. The defining attributes assist in differentiating the occurrence of a specific phenomenon from similar or related ones (Chinn & Kramer 2014:loc 599). A model case was constructed that demonstrated the defining attributes in the central concepts.

The central concept was classified utilising the survey list in Dickoff *et al.* (1968:415–435): Who is the agent? Who is the recipient? What is the procedure? What are the dynamics? What is the context? and What is the outcome?

### Step 2: Placing the concepts in context

The relationship statements will place the concepts in context with one other (Chinn & Kramer 2014:loc 606).

### Step 3: A description of the conceptual framework

The conceptual framework was described in terms of an overview, structural description and the process of the conceptual framework (Chinn & Kramer 2014:loc 606).

### Step 4: Evaluation of the conceptual framework

The evaluation criteria of Chinn and Kramer (2014:loc 606) were used to evaluate the described conceptual framework.

## Ethical considerations

Ethical approval was obtained (Reference number 46/07) from the Faculty of Health Sciences Academic Ethics Committee as well as the Higher Degree Committee, Faculty of Health Sciences. This study was conducted in accordance with the international ethical standards outlined by the Declaration of Helsinki (Holloway & Wheeler 2010:56–57; World Medical Association 2008:2). The ethical principles of respect for human dignity, beneficence and justice were applied (Gerrish & Lacey 2010:28–32; Holloway & Wheeler 2010:59–61) and confidentiality was maintained throughout the study. Voluntary participation was applied as required by the Declaration of Helsinki and participants were informed of their freedom to withdraw from the study at any time.

### Potential benefits and hazards

Potential risks involved emotional discomfort of participants whilst sharing their experiences. The researcher was prepared to provide emotional support and referral to counsellors when needed. The benefit was that a conceptual framework was developed to enhance future support for SNs working with persons with intellectual disabilities.

### Recruitment procedures

Participation was voluntary and measures were taken to ensure that no coercion was involved. The researcher clarified her role as such to the participants. Participants were made aware that they could withdraw from the study at any time.

### Informed consent

Informed consent was obtained from participants before data were collected. The benefits and risks of partaking in the study were disclosed to participants.

### Data protection

Data were safely locked up in a steel cupboard for five years after the study was completed.

## Trustworthiness

Trustworthiness was ensured by applying the criteria of credibility, transferability, dependability, confirmability and authenticity (Gerrish & Lacey 2010:139; Holloway & Wheeler 2010:302–304). Credibility was maintained through the triangulation of data collection methods as well as person triangulation. The researcher applied more than one data collection method by using focus group interviews, naïve sketches, drawings, reflective journals and a reflective letter to collect data from participants. Person triangulation means that data were collected on different levels from the participants. The researcher applied this by utilising different levels such as writing, drawing and voicing the SNs’ experiences. Credibility was enhanced by using reflexivity. The researcher reflected on the research by taking field notes, such as personal notes, to minimise possible biases. Transferability was increased by discussing the demographics of the target population. The results of the research were discussed in depth with supporting direct quotations from the interviews as well as drawings made by participants. Dependability was addressed by code-recoding (consensus with an independent coder) of data and triangulation of data collection methods. Confirmability was achieved by applying objectivity during data collection and analysis. Objectivity was enhanced by utilising an independent co-coder during data collection and independent checking by two supervisors (who are experts in qualitative research) during the research process. Authenticity was reached by active listening to explore the feelings of the SNs with regard to working with persons with intellectual disabilities. The researcher used verbatim transcriptions and direct quotations to ensure a true reflection of the SNs’ experiences.

## Results

Findings from the fieldwork in ‘Step 1: Concept analysis, identifying the central concept’ indicated that SNs initially experienced intense emotional discomfort whilst working with persons with intellectual disabilities. SNs felt emotionally and physically drained, overwhelmed and had a great need to escape from the work situation. SNs experienced emotions of anger, sadness, fear and happiness whilst working with persons with intellectual disabilities. During this time, the SNs are faced with a choice to engage on a deeper emotional level with the persons with intellectual disabilities or to stay distant and aloof. They struggled to adjust and cope whilst working with persons with intellectual disabilities (Janse van Rensburg *et al.* 2012:764–765). A need was identified for additional support for SNs during placement at facilities for persons with intellectual disabilities. The conceptual framework was developed to provide guidance for psychiatric nurse educators during clinical accompaniment for these SNs. The main concept of the conceptual framework – the facilitation of engagement on a deeper emotional level of SNs working with persons with intellectual disabilities – was identified from the qualitative data (discussed under data collection) regarding SNs’ experiences working with persons with intellectual disabilities and classified by using the survey list of Dickoff *et al.* (1968:415–435). The list included the agent, recipient, procedure, dynamics, context and terminus as expressed below:

- The agent is a person who performs the activity of the process of engagement: the nurse educators who will be providing clinical accompaniment through engagement with the SNs, as well as these SNs who will be engaging with the persons with intellectual disabilities are the agents.
- The recipient is the person(s) who will benefit from the facilitation of the process of engagement on a deeper emotional level. The SNs working with persons with intellectual disabilities will benefit through increased emotional support and the persons with intellectual disabilities will benefit when they receive holistic nursing care.
- The context where the process of engagement on a deeper emotional level was facilitated included two contexts. The first context involves the rehabilitation facility for persons with intellectual disabilities where SNs were placed for practical exposure. The second context will refer to the higher educational institution (university) where the SNs are doing training, as well as the nurse educators who are doing clinical accompaniment for the university.
- The procedure refers to the techniques, procedures and/or protocol associated with the facilitation. It will be implemented through a relationship, working and termination phase between the nurse educators and SNs.
- The dynamics that motivate the facilitation refer to the agents, recipients and resources. The facilitation of engagement on a deeper emotional level will facilitate a process of personal transformation that will lead SNs to discover meaning of the self. The initial emotional discomfort experienced by the SNs whilst working with persons with intellectual disabilities will be transformed into an opportunity for growth, enhancing their mental health through identifying their emotional processes. The nurse educators will enhance their clinical accompaniment practices as a resource for SNs.
- The terminus is the end result of the process. The SNs’ process of personal transformation and the start of a journey towards the discovery and internalisation of meaning whilst working with persons with intellectual disabilities, will contribute to the outcome. The outcome will be the facilitation of the mental health of SNs working with persons with intellectual disabilities.

The essential and related attributes of the concept, ‘the facilitation of engagement on a deeper emotional level’ of these SNs were used to construct relationship statements. The relationship statements on ‘engagement on a deeper emotional level’ referred to engagement with these SNs by the nurse educators as well as engagement by these SNs with the persons with intellectual disabilities.

The following relationship statements have been identified in this conceptual framework. The nurse educators provide support to SNs whilst placed in the clinical learning environment. Support can be rendered by the nurse educators by engaging with SNs whilst they are in practice to explore their emotional discomfort. A trusting relationship is formed to enhance support. Coping and adjustment of SNs are explored and strengthened through mobilisation of resources in both the internal and external environment of the SNs to promote their mental health. SNs engage on a deeper emotional level with persons with intellectual disabilities after a process of self-discovery. This initiates the process of personal transformation through self-reflection.

The relationship statements among concepts highlighted the understanding of the phenomenon.

## An overview of the conceptual framework

The conceptual framework will serve as a framework of reference for the facilitation of a process of engagement on a deeper emotional level by SNs working with persons with intellectual disabilities. The mental health of the SNs will be promoted through the process of engagement on a deeper emotional level, as SNs will mobilise resources in their internal and external environments. The mental health of these SNs will also be promoted as they will start a journey towards self-discovery.

**FIGURE 1:**
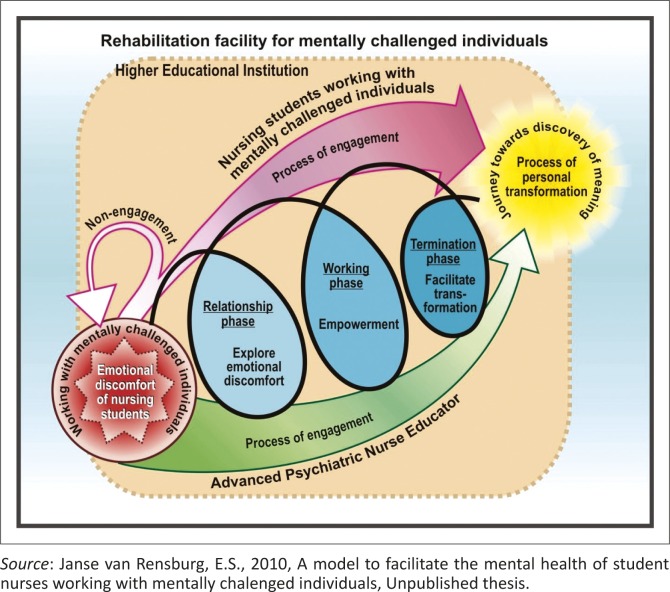
Janse van Rensburg's conceptual framework (2010) to facilitate the mental health of student nurses working with persons with intellectual disabilities.

The conceptual framework for the facilitation of a process of engagement on a deeper emotional level for these SNs is reflected in Figure 1. The process of facilitation will involve a relationship, working and termination phase between the psychiatric nurse educators and the SNs working with persons with intellectual disabilities. The facilitation of a process of engagement will move these SNs from a position of initial emotional discomfort towards a process of personal transformation.

## The structure of the conceptual framework

The structure of this framework will be discussed by referring to the discussion by Janse van Rensburg *et al.* (2012:393–412). The components of purpose, assumptions, context and theoretical definitions are addressed in describing the structure of the conceptual framework (Chinn & Kramer 2014:loc 599, 606).

## Purpose

The purpose of the conceptual framework is to serve as a framework of reference for the nurse educators during clinical accompaniment of SNs and to facilitate a process of engagement on a deeper emotional level for SNs working with persons with intellectual disabilities.

## Assumptions of the conceptual framework

The ‘Theory for health promotion in nursing’ (University of Johannesburg 2009:4) provided assumptions for the conceptual framework as part of Phase 2. The results from Phase 1 and the literature review were applied in the assumptions to guide Phase 2.

Facilitation of engagement on a deeper emotional level implies the empowering of the SNs to mobilise resources in their internal and external environments. Within the internal environment the SNs explore the choice to engage in emotional commitment with the persons with intellectual disabilities.

Engagement on a deeper emotional level involves experiencing and expressing feelings of anger, sadness, fear and happiness whilst working with these individuals.

The facilitation of a process of engagement on a deeper emotional level will take place during a relationship, working and termination phase. The SN is seen as interacting holistically with the environment. This interaction will be in an integrated manner through group support and supervision (University of Johannesburg 2009:4). The integrated manner of interaction will include engagement on a deeper emotional level for these SNs.

The environment refers to the internal and external environments of the SNs. The internal environment consists of three dimensions: body, mind and spirit. The external environment consists of the physical, social and spiritual dimensions (University of Johannesburg 2009:4). The spiritual dimension refers to the values and religious aspect in the external environment (University of Johannesburg 2009:4).

Mental health promotion includes the process of mobilising resources, which will refer to the process of personal transformation and the start of a journey towards self-discovery. Self-discovery included assessment of self-growth and generating an appreciative attitude.

## The theoretical definitions of concepts

The central, essential and related concepts have been developed to provide conceptual clarity and application to the context of this study.

The facilitation of engagement on a deeper emotional level by the psychiatric nurse educators entails a process of empowering SNs. Empowerment includes the mobilisation of resources in the promotion of mental health by demonstrating emotional commitment. Emotional awareness is created through the experiencing and expressing of emotions of anger, sadness, fear and happiness whilst working with persons with intellectual disabilities.

### Mobilising resources

Mobilise resources refer to resources in the internal and external environments of SNs working with persons with intellectual disabilities. Within the internal environment, it refers to the body, mind and spiritual dimension. Resources were mobilised in the internal environment by providing support and creating a safe space where self-awareness was enhanced through reflection. The external environment refers to the physical aspects at the facility. This can include structural resources in caring for these individuals, for instance wheelchairs, beds and bath facilities. The social dimension includes social support from other caregivers or students. The internal and external environments were discussed under the assumption that they were part of the structure of the conceptual framework.

### Promotion of mental health

Promotion of mental health will refer to the realisation of potential for emotional growth of these SNs. Mental health relates to the ability of the SNs to move through a process of personal transformation, as well as the ability to start a journey towards self-discovery as SNs. The promotion of mental health might also refer to the ability of SNs to adjust to this challenging working context and may include the ability to experience and express feelings of anger, sadness, fear and happiness whilst working with these intellectually disabled individuals.

### Process of personal transformation

The process of personal transformation can be divided into emotional, spiritual and interpersonal enrichment of SNs working with these individuals. The process of personal transformation was discussed under the assumptions as part of the structure of the conceptual framework.

### A journey towards the discovery of meaning

The process of personal transformation created a context for the SNs’ self-discovery. The meaning captured the choice they made of whether to engage on a deeper emotional level with the persons with intellectual disabilities. The journey towards self-discovery was discussed under the assumptions as part of the structure of the conceptual framework.

### A structural and process description of the conceptual framework

The conceptual framework, as displayed in Figure 1, will be discussed in terms of the meaning and interaction between parts of the conceptual framework. The structure of the conceptual framework consists of the:

- emotional discomfort of SNs working with persons with intellectual disabilities,
- process of engagement of the nurse educators and these SNs, and
- process of personal transformation and a journey towards SNs’ self-discovery.

### Emotional discomfort of student nurses working with persons with intellectual disabilities

During the initial interaction with these individuals, the SNs experienced intense emotional discomfort which is linked to the lack of physical and emotional boundaries; the physical aspects of the working context. The emotional discomfort is illustrated in red in Figure 1 and represents a situation where the SNs felt overwhelmed and could not escape. It can reflect the ‘fight or flight’ response they may be experiencing.

### The process of engagement on a deeper emotional level of the nurse educators and student nurses

The conceptual framework indicates two role players: nurse educators and SNs. The process of engagement on a deeper emotional level was divided into a relationship, working and termination phase. The arrow representing the nurse educators was green in Figure 1. Green can represent emotional healing, protection, a sense of self-control, and harmony. It alleviates anxiety. The intensity of the colour differs to represent the level of engagement as more intense during the relationship phase when the nurse educators’ level of engagement is at its highest. The colour chosen to represent the SNs in Figure 1 is pink. Pink is associated with the sweetness of youth as well as fragility. The intensity of the colour differs to represent the level of engagement of the SNs with the nurse educators and the persons with intellectual disabilities. The colour is the most intense during the termination phase when the SNs’ level of engagement is at its highest.

The green arrow, representing the nurse educators in Figure 1, is placed below the phases and arrow representing the SNs. This placement represents the supportive role the nurse educators play during the process of facilitation of engagement on a deeper emotional level for nurse educators.

The role of the nurse educators is to role model self-control and harmony to the SNs. They need to create a context of trust and safety to enable the SNs to explore and express their experiences of working with persons with intellectual disabilities. SNs need a safe environment where they can share challenges and concerns whilst working with these individuals. The process of engagement involves a relationship, working and termination phase. The colour blue was chosen for the relationship, working and termination phases within the process of engagement (Figure 1) as it symbolises trust, inspiration and wisdom. Blue represents characteristics of trustworthiness, dependability, commitment and healing. Within this conceptual framework, blue will signify emotional commitment and knowledge of the SNs within the process of engagement on a deeper emotional level with the nurse educators as well as the persons with intellectual disabilities. The intensity of the colour in the phases also differs as the emotional commitment and knowledge of the SNs increase and intensifies.

### Relationship phase

During the initial process of engagement, the level of engagement of the nurse educators is high. The green arrow in Figure 1 has a broad basis at the emotional discomfort that grows thinner as it moves through the relationship phase towards the working phase. The intensity of the colour also becomes lighter as it is linked to the width of the arrow, representing the intensity of the engagement. The nurse educators demonstrate commitment and compassion towards these SNs. A therapeutic relationship is established with the SNs by creating a context of trust, creating rapport and demonstrating empathy and acceptance. The nurse educators will convey empathy and acceptance within a context of trust and demonstrate compassion and acceptance. They will explore and identify the emotional discomfort experienced by SNs. SNs will have an increased awareness of their emotions and the management thereof. They will be faced with the choice of engagement or non-engagement on a deeper emotional level with these individuals. The level of engagement of the SNs with the nurse educators and the persons with intellectual disabilities is low within the relationship phase. The SNs are still entering into a therapeutic relationship with the nurse educators and trust might still need to be developed. During this phase, the SNs may not experience commitment or compassion towards these individuals. SNs are faced with a choice of engagement or non-engagement with the persons with intellectual disabilities. SNs who choose non-engagement return to emotional discomfort and do not move forward to the working phase. They tend to stay emotionally distant and aloof.

Engagement at a deeper emotional level with these SNs will be facilitated by conducting therapeutic group discussions with these students. The nurse educators will take on the role of group therapists and facilitators and they will respect the SNs’ freedom of choice with regard to engagement or non-engagement at a deeper emotional level with persons with intellectual disabilities, and assist those SNs who choose engagement at a deeper emotional level to move to a process of personal transformation.

During the therapeutic group discussion, the nurse educators will encourage direct interaction among group members. The therapeutic factors of group therapy will be applied, specifically universality and creating a sense of belonging of group members (Yalom 2005:107).

### Working phase

During the working phase, the nurse educators’ level of engagement is moderate. They act as role models of the ‘gift of self’ during the relationship and working phase. The ‘gift of self’ is defined by Vanier (1988:6) as follows: ‘In some mysterious way, they [*caregivers*], too, are giving life, love, light, and peace to others around them. I am constantly touched by the way very humble, fragile people, through trust and love, can tap the well of living waters in others, calling forth the gift of self.’ The nurse educators empower the SNs with knowledge and an awareness of their emotional process. They facilitate group discussions to create a sense of universality and belonging among SNs with regard to working with these individuals. As SNs become more empowered and engaged, the nurse educators’ level of engagement with SNs, on a deeper emotional level, decreases. This is indicated by the lighter colour and decrease in width of the green arrow. During the working phase the level of engagement of the SNs students is moderate. The level of engagement can refer to the engagement on a deeper emotional level with the nurse educators as well as with the persons with intellectual disabilities.

The process of engagement at a deeper emotional level will involve the empowerment of SNs. Empowerment, in the research on which this article was based, focused on the following three concepts:

- Empowering the SNs with knowledge and self-awareness of their emotional process during exposure to the demanding context.
- Empowering the SNs to mobilise resources in their internal and external environments.
- Involving the SNs in experiencing and expressing feelings of anger, sadness, fear and happiness whilst working with persons with intellectual disabilities.

The nurse educators’ level of engagement with SNs, as well as the SNs’ engagement with the nurse educators is on a moderate level. During this phase the nurse educators engage at a deeper emotional level with SNs by demonstrating empathy for them. They will acknowledge the SNs’ challenges in working with persons with intellectual disabilities, which may include physical and emotional challenges.

Empathy can create an awareness of the self. The nurse educators will facilitate self-awareness of the SNs by exploring their emotions. As empathy and self-awareness increase, the facilitation of engagement at a deeper emotional level is established. The empowerment process will involve the creation of a positive environment where the SNs are assisted in mobilising resources within their internal and external environments. ‘Mobilisation of resources’ refers to utilising the opportunities that promote mental health and identifying and overcoming obstacles in the promotion of mental health (University of Johannesburg 2009:4).

Resources in the internal environment of the SNs working with persons with intellectual disabilities include dimensions of the body, mind and spirit.

SNs’ dimension of the body may refer to the physical ability and the strength they may need to display whilst working with persons with intellectual disabilities. This ability can assist them in providing care and assisting with hygienic needs. The dimension of the mind may refer to the intellectual, emotional and volitional processes. The intellectual process includes critical thinking and the processing of information. The emotional dimension includes the emotions the SNs may be experiencing whilst working with persons with intellectual disabilities. These emotions were mentioned earlier. Volition relates to the decision-making process as well as the execution of a choice by the SNs. It refers to their decision regarding engagement or non-engagement at a deeper emotional level with these individuals (University of Johannesburg 2009:4). The spiritual dimension refers to the SNs’ relationship with God.

Resources in the external environment include physical, social and spiritual dimensions (University of Johannesburg 2009:4). ‘Physical’ refers to the external structures in the environment. This includes the rehabilitation facility where the SNs are placed and refers to the physical facilities and resources. Physical resources include the facilities in terms of bathrooms and changing facilities, sleeping areas, kitchen and dining areas, and relaxation areas. This also refers to stimulation programmes and physiotherapy activities. The social dimension refers to human resources in the external environment. This includes staff members, their level of training and commitment to their work. It may also include the nurse-patient ratio between persons with intellectual disabilities and staff members. The spiritual dimension refers to values and religious aspects in the external environment. It may refer to the SNs’ religious denomination, as well as to support groups within the church and religious activities.

The nurse educators need to explore the available resources in the internal and external environment of the SNs and empower the SNs with regard to identifying and expanding on existing resources.

The main focus of the nurse educators will be on the facilitation of the process of engagement at a deeper emotional level of the SNs with regard to themselves as well as persons with intellectual disabilities. As these SNs engage at a deeper emotional level with these individuals, the nurse educators’ engagement with the students will decrease. This fosters independence within the SNs.

As the deeper emotional level of engagement increases, the SNs become more committed and compassionate towards these individuals. SNs’ self-awareness increases and they experience and show more empathy towards the self and others. The SNs identify and express emotions of anger, sadness, fear and happiness, but as they become more empowered, they become more engaged on a deeper emotional level with the nurse educators as well as the persons with intellectual disabilities.

### Termination phase

During the termination phase, as the nurse educators facilitate transformation and the start of a journey towards the discovery of meaning, their deeper emotional level of engagement with SNs decreases. Their relationship with the SNs reaches a stage of ending and ensuring that the SNs will grow to independence through their process of personal transformation. During the termination phase a process of personal transformation is facilitated for the nurse educators and they also start a journey towards the discovery of meaning for themselves as nurse educators. The SNs also experience a high level of engagement on a deeper emotional level with the persons with intellectual disabilities at the termination phase. They may differ with regard to their engagement with the nurse educators as they strive towards emotional independence.

During the termination phase, the facilitation of a process of personal transformation will be explored and facilitated. This process can be divided into emotional, spiritual, and interpersonal enrichment of the SNs working with persons with intellectual disabilities.

The SNs experience an unexpected change in their attitude towards physically and mentally challenged individuals, and they feel inspired, motivated and enriched by their experiences with these individuals.

The nurse educators will create a context of self-reflection for SNs by encouraging them to write self-reflective journals on their experiences of working with persons with intellectual disabilities.

The nurse educators can furthermore facilitate the ‘gift of self’ by role-modelling it during their engagement with the SNs. The ‘gift of self’ also links with the journey towards the discovery of meaning. The focus shifts from the initial differences between these individuals and the SNs, towards the similarities. The SNs will start looking for the persons behind the intellectual disabilities. During this shift they discover meaning as their lives expand beyond themselves and their own needs towards the needs of these individuals. The shift inspires the SNs to open their hearts and start giving of themselves.

The process of personal transformation will create a context for the SNs to start a journey towards the discovery of meaning for the self. The nurse educators may demonstrate that meaning is always present in challenging contexts; it only needs to be discovered to create opportunities for the future.

The SNs become aware of their personal process of transformation with regard to emotional, spiritual and interpersonal enrichment. Discussions of personal growth and self-reflection in journals assist with the process of personal transformation. This leads them to start the journey towards the discovery of meaning within the relationship with the self, others and the environment.

### Process of personal transformation and a journey towards meaning of student nurses working with persons with intellectual disabilities

Personal transformation transpires on an emotional, spiritual and interpersonal level of enrichment. The discovery of meaning links to the facilitation of mental health. SNs start mobilising their resources in their internal and external environments that facilitate, promote and restore their good mental health as support is generated. As an end result, the relationship with the self, others and the environment is enriched.

## Discussion

### Outline of results

The main aim of this study was to develop and describe a conceptual framework for nurse educators to provide support and guidance to SNs to enable them to adjust and cope whilst working with persons with intellectual disabilities. The contribution of this article lies in the relevance of this conceptual framework for these SNs and the nurse educators providing support to student nurses in the form of clinical accompaniment to enhance their professional and personal development. The first objective, which explored and described the experiences of SNs taking care of persons with intellectual disabilities, was addressed in a previous publication (Janse van Rensburg *et al.* 2012:761–769). The focus of this article was to address the second objective by describing a conceptual framework to facilitate the mental health of SNs taking care of these individuals. The conceptual framework was evaluated using Chinn and Kramer’s evaluation criteria, namely: clarity, simplicity, generality, accessibility and importance (Chinn & Kramer 2014:loc 606). The evaluation indicated that the conceptual framework was clear, understandable and played an important role in generating support for students working with mentally challenged individuals.

### Practical implications

This study contributes a conceptual framework that can be practically applied during clinical supervision of SNs working with persons with intellectual disabilities. It can provide guidance to nurse educators to enrich their practices of supervision. In turn, this may improve SNs’ professional identity and personal growth, improving their mental health and practices of care for these individuals.

## Limitations of this study

Only two focus groups’ interviews were used to collect data, which might have limited the amount of data. However, multiple methods of data collection were included to increase the depth of the data. These methods included naïve sketches, reflective journals, a reflective letter and field notes to triangulate the data obtained from the interviews with the two focus groups. Only one participant was male. The question can be asked as to what the outcome would be if more males participated. In South Africa, at present, few males choose the nursing profession. A suggestion can be made for future research to include more male participants during the implementation and evaluation of the conceptual framework.

## Recommendations

Recommendations were made on nursing education, practice and research. This conceptual framework can be utilised in nursing education as part of clinical accompaniment to SNs. The role of the nurse educators can be enhanced to generate more effective support for SNs working with persons with intellectual disabilities and to assist students with personal and professional growth. It can broaden the subject knowledge with regard to how SNs can be empowered to manage their emotional process in terms of exploring and expressing emotions in challenging contexts within the nursing practice. This can lay the groundwork for future management of emotions experienced in a challenging context that may lower the incidence of exhaustion or emotional burnout within the nursing profession. The conceptual framework can inspire SNs to make a difference in nursing practice.

It is still unclear what the experiences of nurse educators are in the clinical accompaniment to SNs working with persons with intellectual disabilities. Future research might shed light on these experiences and the contributions from the perspective of the nurse educators on clinical accompaniment of SNs working with persons with intellectual disabilities. It is envisioned that a post-doctoral study will follow to implement and test the current conceptual framework to facilitate the mental health of SNs working with persons with intellectual disabilities. Thereafter, the conceptual framework can be adjusted and implemented in other challenging contexts in nursing. Data can be collected during the implementation, evaluation and adjustment of the conceptual framework and incorporated in articles.

## Conclusion

SNs working with persons with intellectual disabilities experience emotional discomfort, which may increase work-related stress. Increased stress might negatively influence their adjustment and coping abilities whilst working with these individuals. Therefore, working with persons with intellectual disabilities could negatively influence their mental health. The nurse educator plays an important supportive and facilitative role in providing support and enhancing the learning experience of SNs during clinical accompaniment.

The main aim of this article was to describe a conceptual framework for the nurse educators to use in order to facilitate the mental health of SNs working with persons with intellectual disabilities. Within the context of SNs enrolled for a degree in nursing science at a university, nurse educators provide clinical accompaniment in the psychiatric learning environment and should have a master’s degree in advanced psychiatric nursing science (advanced psychiatric nurse educator). The conceptual framework was written as a contribution for the nurse educators to use in order to provide effective support for these SNs. Effective support assists SNs in their adjustment and coping, thus in facilitating their mental health. The process of engagement moves the students from a place of emotional discomfort to a process of personal transformation, which starts a journey towards the discovery and internalisation of meaning within the self-identity of the SN. One of the students in the research project on which this article is based reflected on her discovery of meaning by writing in her naïve sketch:

… it has opened up more opportunities for me to realise the speciality I would like to enter into. Winston Churchill said, ‘We make a living by what we get but we make a life by what we give.’ And just my giving of all that I could to these children, I have made my life more worthwhile.

To conclude, this conceptual framework can be applied to SNs in any challenging context that might create emotional discomfort for them. The researcher is in the process of implementing the conceptual framework to support SNs working with persons with intellectual disabilities.
